# Supplementary material for: Genomic diversity in Porphyromonas: evidence of Porphyromonas catoniae commensality in lungs
Source: Microb Genom. 2025 Jun 13;11(6):001411. doi: 10.1099/mgen.0.001411 (PMC12166133; doi:10.1099/mgen.0.001411)
Supplement: Uncited Fig. S1. [file mgen-11-01411-s001.pdf]

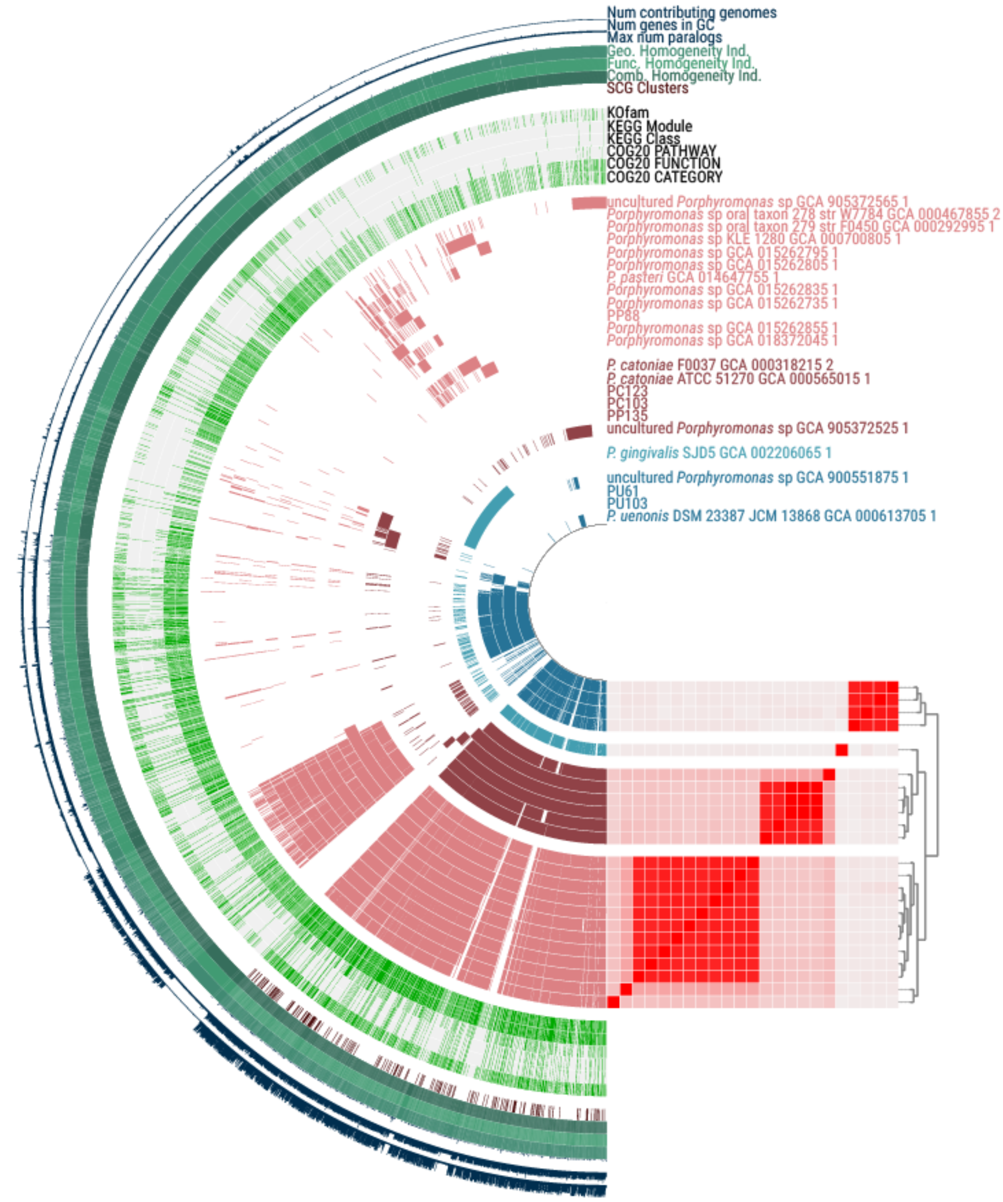

Supplementary Figure 1. Pan-genome analysis of pulmonary strains of *Porphyromonas*.
PC103, PC123, PC135, PP88, PU61, and PU103 were isolated from the lungs of people with cystic fibrosis. Other genomes were downloaded from NCBI.
